# Reading Disorders: Pro-Eating Disorder Rhetoric and Anorexia Life-Writing

**DOI:** 10.1353/lm.2016.0023

**Published:** 2016

**Authors:** Emma Seaber

**Keywords:** Anorexia nervosa, memoir, discourse, pro-anorexia, material culture

## Abstract

This article explores the relationship between eating disorders and reading behaviors, arguing that there is a meaningful difference in a minority of readers' approach to and understanding of anorexia life-writing, and of literary texts more broadly. To illuminate this distinction, this article begins by considering the reported deleterious influence of Marya Hornbacher’s anorexia memoir, *Wasted*, elaborating the ways Hornbacher offers a positive presentation of anorexia nervosa that may, intentionally or not, induce certain readers to “try it” themselves. This is followed by an exploration of how Hornbacher’s own reading praxis is implicated in a discursive feedback loop around anorexia narratives. It concludes with a discussion of disordered reading attitudes in relation to the emergence of the “pro-anorexia” phenomenon.

Anorexia nervosa, Joan Jacobs Brumberg argues, “has become *au courant*, an ‘in’ disease among affluent adolescent and young adult women,” its typical sufferer “intelligent, attractive, polite, demanding of herself.”^1^ These words echo Elaine Showalter’s description of melancholia as “a *prestigious* disorder of upper-class and *intellectual* men.”^2^ Indeed, just as melancholia developed as a cult phenomenon among well-to-do men between the seventeenth and nineteenth centuries, so anorexia nervosa has been seen as a fashionable, albeit underground, “lifestyle choice” amongst young women over the last two decades. Although the incidence of anorexia nervosa has been increasing since its identification in the 1870s, and particularly since the mid-twentieth century, it is only since the late 1990s and the advent of widespread Internet access that anorexia has seemed to transform conspicuously from an isolating, individualist disease to, as many view it, a communal activity and group identity. Pro-anorexia websites, which offer *soi-disant* tricks and tips to avoid eating or being found out and which recast the condition as an elite, ascetic lifestyle or even a religious cult, have generated widespread and enduring media concern, “revulsion [and] . . . morbid fascination.”^3^ On these sites, anorexia becomes “Ana,” a goddess figure to whom acolytes are encouraged to devote themselves; whose “thin commandments” they must follow; and whom they worship “with soul . . . heart and . . . bodily functions.”^4^ For many, one book lies at the core of their devotion: Marya Hornbacher’s *Wasted: A Memoir of Anorexia and Bulimia*. Published in 1998 and written when Hornbacher was twenty-three, this text was part of a new trend in the United States for bold, provocative, and sometimes lurid accounts of young women’s psychiatric crises, ushered in by such memoirs [Other P-484] as Susanna Kaysen’s *Girl, Interrupted* (1993) and Elizabeth Wurtzel’s *Prozac Nation* (1994).

Many anorexia nervosa patients describe using *Wasted* to exacerbate their anorexic thoughts and behaviors. Nicole Johns, in her own eating disorder memoir, *Purge*, calls it the “preeminent” memoir, the “eating disorder bible,” describes its “cult following” amongst eating-disordered patients, and writes that “women attempted to smuggle *Wasted* into the treatment facility.”^5^ Similarly, reviews of Hornbacher’s memoir on bookseller Amazon’s website caution, “don’t say you weren’t warned”; “it is toxic to people who are sick”; it “is NOT a good book to give to a teenage girl or any person with an eating disorder.”^6^ Whilst it may seem a spurious suggestion that a book might trigger or encourage an eating disorder, Hornbacher herself explains how, upon reading *The Best Little Girl in the World*, Steven Levenkron’s “rather romanticized” novel about anorexia nervosa, she “wanted to be [that girl]: withdrawn, reserved . . . wholly absorbed in her own obsession.”^7^ It is also not the first time such a claim has been leveled at a memoir written “to keep people from going where I went.”^8^ During the 1820s, for instance, Thomas De Quincy’s *Confessions of an English Opium-Eater*, presented as a “useful and instructive” record of the author’s addiction and the “horrors of opium,” received criticism for encouraging its readers to try opium themselves.^9^ Going back a little further in time, we encounter the themes explored in other essays in this collection—suggestions that reading about particularly emotive subjects might prove a singularly harmful exercise, particularly for adolescent girls. For instance, the suggestion that Samuel Richardson’s 1747–48 novel *Clarissa*—wherein the eponymous heroine willfully starves herself to death and has been described by Maud Ellmann as a “literary anorectic”—harmed young women readers by “discomposing their stomachs” presents a curious similarity to contemporary concerns about the dangers of reading accounts of self-starvation.^10^

Certainly, it would be specious to posit that Hornbacher’s book is capable of spontaneously causing anorexia nervosa—it has not been established that any text can trigger the disorder in healthy individuals—but there does appear to be a special relationship between particular writing and reading practices and anorexia identity formation and maintenance for some readers with a predisposition to eating disorders. Certain experiences and accounts of anorexia (as an illness, as a philosophical or political position, as a lifestyle) seem to rely upon peculiar ways of approaching, producing, and consuming written texts, and both anorexia memoirs and the pro-ana phenomenon [Other P-485] reveal modes of reading—interpreting, understanding, and responding to—social and cultural texts that are highly distinctive.

## Beyond “Mirror, mirror”: Rereading Anorexia Nervosa

Currently, anorexia is widely considered a sort of visual condition. The terms used to write about the disorder focus on sight, image, perception, looks, mirrors: we talk about people “looking anorexic”; our critical discussions center around obvious thinness and body image ideologies; we illustrate anorexia by showing someone gazing in a mirror and seeing a distorted reflection; and we witness and contribute to innumerable debates about how the ubiquitous visibility of thin celebrities (or models, or Barbie dolls) must be responsible for the increasing incidence of anorexia. This view sees anorexia as a kind of mimicry: the anorectic has seen thinness around her and attempted to reflect those images corporeally. Reflexively, we reach towards motifs from well-known stories to frame the disorder in these terms: even support websites and charitable organizations, and of course pro-anorexia websites, take names like “The Looking Glass” and “Mirror Mirror.”^11^ The underlying assumption, therefore, is that anorexia is a condition of superficiality, or of vanity. This understanding is inadequate for several reasons.

This view propels pervasive beliefs about the influence of mass media on anorexia incidence or on “prospective” anorexia patients (i.e. teenage girls, in the main): the view, as Michele Crisafulli and colleagues put it, that anorexia nervosa is “a purely sociocultural phenomenon in which young girls succumb to the image of ideal beauty put forth in fashion magazines.”^12^ In this way the anorectic is held solely to blame for having become ill, yet her illness is also seen as an apparently inevitable product of visual culture (either way absolving anyone else of guilt or responsibility). Evidence suggests that this view of anorexia as volitional is quite common, and that it has a stigmatizing and ultimately harmful effect on attitudes towards those with anorexia. Several studies have highlighted the negative attitudes health care professionals hold towards anorexia patients, reporting, for example, that such patients are less liked, finding the prevailing opinion among caregivers is that anorexia nervosa is self-inflicted and willful, and that patients “have only themselves to blame” for their condition.^13^ Public attitudes towards patients with anorexia nervosa are similarly negative.^14^ Research by Arthur Crisp found that patients [Other P-486] with anorexia were frequently considered to blame for their condition and thus should be able to “pull themselves together.”^15^ In Crisp’s study, feelings of blame towards anorexia patients far exceeded those for depression or schizophrenia and approached levels directed at those with drug and alcohol dependence. This indicates a high level of public stigmatization of eating disorder patients. These studies are just part of a larger body of research which suggests that the view of anorexia patients as too-credulous (and implicitly vacuous) consumers of mass media contributes to attitudes of blame and stigmatization.

A further problem with the visual model for conceiving of anorexia nervosa is that it leaves little room for alternative narratives, thereby marginalizing accounts of anorexia that do not fit this framework. Portraying anorexia as a so-called slimmer’s disease or as a celebrity copycat phenomenon among teens has meant that anorexia is increasingly not recognized when it presents differently. Recent work has found a significant portion of anorexia cases emerge without volitional weight loss or any attempt at dieting. In these cases, patients develop anorexia nervosa having unintentionally lost weight (thanks to a parasitic infection, say, or cancer treatment).^16^ For these patients, and for others who in one or more ways challenge the predominant view—for boys and men, for older women, for people of color—their illness becomes doubly aberrant: since the dominant narrative of anorexia has converged on this teen girl/visual model, other narratives, other anorectics, become invisible, yet these patients are nevertheless subject to the same abiding social stigma that assaults all anorexia patients. Anorectics whose eating disorders are not accommodated by the prevailing narrative are less likely to seek help and are less likely to have their problems recognized as such if they do.^17^ Delays in diagnosis and treatment have known negative consequences for illness duration and recovery trajectory.^18^ Any anorectics for whom the visual model does not seem to fit suffer more and for longer while that model persists in determining the thrust of popular eating disorders discourse.

Finally, this predominant conceptual position on anorexia as a visual phenomenon requires a concerning and persistent focus on the strictly visual-corporeal elements of the condition. That is, seeing anorexia as the pursuit of socially idealized thinness yields treatment narratives which focus on correcting extreme thinness, holding reversal of emaciation as the primary marker of health. Using weight as the main measure of illness severity leads to enormous gaps in care provision (and patients often needing to lose weight in order [Other P-487] to qualify for treatment). Helen Gremillion has further criticized this microscopic focus on the body, arguing that the “constant surveillance and manipulation of the body on a very intimate scale” (for instance, daily weight monitoring to 100-gram precision), could very well make hospitalizations longer and more harmful and contribute to increasing illness chronicity in anorexia nervosa.^19^ There are thus multiple reasons to conceive of anorexia differently. This is not to say that anorexia is not at all concerned with visual culture or body image—it surely can be—but conceiving of anorexia nervosa as a condition for which interpretation, rather than perception—reading, not seeing—is a more important factor may begin to answer, in a way that other conceptual models are unable to, some of the questions raised by the growth of pro-anorexia texts and the charge that anorexia may be a textually transmitted disease.

Several studies that I discuss below—including Ancel Keys’s pioneering Minnesota Starvation Experiment and others examining the effects of viewing pro-eating disorder websites—emphasize a relationship between disordered modes of eating (for example not-eating or binge-eating) and disordered modes of reading, whereby innocuous or cautionary texts are co-opted, take on new meaning, or are transformed into compelling role models. The Minnesota Starvation Experiment was a 1944–1945 medical study designed to investigate the physical and mental effects of extreme caloric restriction and dramatic weight loss and to assess the effects and effectiveness of weight restoration strategies. Participants were recruited from a pool of conscientious objector volunteers in the Civilian Public Service. The healthy male participants had their caloric intake reduced by half for twenty-four weeks. Over the course of the study, the men exhibited a range of obsessive and compulsive food-related behaviors, including hoarding not only food items but recipes—almost one hundred cookbooks, which they would read obsessively.^20^ In essence, being placed on a restricted diet not only triggered expected physiological changes in participants, it also caused them to experience a range of psychological symptoms similar to those found in eating disorders. These endured even during weight restoration. The participants’ behavior with books suggests that starvation or low body mass affects approaches to texts and reading habits. This connection may begin to inform our understanding of what initiates the processes involved in pro-anorexia reading networks.

More recent studies have examined levels of eating and body image disturbance in those who frequent pro-eating disorder websites and the effects of viewing pro-anorexic web content. Harper, Sperry, [Other P-488] and Thompson found that participants who regularly viewed websites promoting eating disorders had higher levels of eating disturbance.^21^ Anna Bardone-Cone and Kamila Cass found that healthy “participants who viewed a pro-anorexia website had greater negative affect, lower social self-esteem, and lower appearance self-efficacy” following the viewing than those who were exposed to a comparison website.^22^ This research presents a conflicted picture of the factors driving disordered eating, and how, or how far, pro-anorexia might fit into this: Bardone-Cone and Cass’s results imply a direct link between cultural messages and body image and eating attitudes, while Harper, Sperry, and Thompson’s findings note a similar correlation among regular consumers of pro-anorexia discourse, although they do not suggest causality. In any case, widespread concerns about the harmful influence of pro-anorexia seem justified, and these findings reinforce the urgency of questions raised about the origins of anorexia nervosa and the salience of pro-anorexia rhetoric in anorexia illness experience.

We may find clues as to why some people are more susceptible to anorexia nervosa than others by looking more closely at what appears to be a significant relationship between reading particularly (sub)culturally important texts—like *Wasted*—and disordered eating. Noting the wealth of criticism attesting to the anorexogenic influence of Hornbacher’s memoir, one 2006 study measured the memoir’s effects on readers’ eating attitudes and behaviors.^23^ The study, crucially, found that participants—healthy women college students—showed no significant changes across three separate measures of eating behaviors and attitudes after reading the text.^24^ These findings directly contradict testimony from eating disorder patients at a variety of sources, suggesting that there is an interpretative gulf between certain readers’ (and eaters’) approaches to and understanding of the text. Indeed, Jennifer Thomas et al. suggest that readers who “vary in their underlying predispositions for disordered eating may also differ with regard to the salience, encoding, and retrieval of memoir information.”^25^ From a literary standpoint, we might view this as a diversity of interpretative engagement with narrative: vulnerable readers differ in their approach to close reading practices and discursive involvement with texts (especially memoirs). Considered together, the above studies also suggest, in the relationship between reading disturbance and eating and body image disturbance, that there may be a method, peculiar to readers with or predisposed to eating disorders, of interpreting texts in a maladaptive way. Indeed, this way of reading could constitute a precondition for many cases of anorexia nervosa.[Other P-489]

I suggest the term “reading disorder” to describe this phenomenon and activity at work; it denotes the reading (and writing) practices by which textual artefacts—first among them case histories and personal narratives, which are normatively construed as negative and cautionary—are reinterpreted or repurposed as aspirational.^26^ It also denotes the processes by which other texts (for instance novels and self-help books) and other practices (for example studying, writing essays, or even writing an anorexia memoir) transform and are transformed by the anorexia illness experience. Using this conceptual model to describe the peculiar intertwinement of narrative behaviors with anorexia need not impute direct etiology: that is, disordered reading is presented as a measure of propensity, not a measure of causality. Moreover, in the term “reading disorder” I intend to point not only to the relatively direct process of reading as simply receiving (or even consuming) a text, but to hold on to the numerous and complex meanings under the broader sense of the term: “reading” here is a conscious invocation of the literary, and in particular of the hermeneutic: of teasing out arcane, profound, perhaps broadly bewildering meaning—and of the anorexia memoir as scripture. Of anorexia nervosa as a type of close reading, even. Thus, “reading disorder,” as I conceive it, encompasses both the conventional and the multiple, expansive senses of “reading,” to include approaches to, understanding of, engagement with and responses to textual artefacts. Here I would reiterate that disordered reading, rather than being a condition of extreme impressionability, sees readers privilege *their* interpretation of certain textual events over any number of others that may contradict it. This position aligns with Thomas et al.’s suggestion of differences in information salience and encoding across reading behaviors, particularly concerning the subject of anorexia. Presented with a text, the (reading) disordered reader’s analysis will, thus, reflect only their reading of themselves. The reading disorder hypothesis resists and poses a direct challenge to the implicit conclusions of the visual interpretation of anorexia and thereby avoids the negative consequences of framing the illness as disorder of superficiality.

This understanding of disruptive and non-normative responses to textual matter as a type of reading disorder allows, I believe, for closer and keener analysis of the processes and rhetoric behind the growing eating disorder cult and its anorexic discourse—that is, the looping effect illustrated by the synthesis, incorporation, and regurgitation of pro-anorexia content within and between online communities.[Other P-490]

This paper, then, is concerned with life-writing rhetoric, anorexia as a reading disorder, and the development of anorexic discourse. In the following analysis, I explore links between disordered eating and disordered reading and examine pro- anorexia as an alternative interpretative mode, outlining and analyzing the rhetorical mechanisms at work within the discourse that reveal anorexia as a reading disorder and anorexia memoir narratives as particularly revelatory in this understanding. Considering texts and their reception and deployment in this manner will offer a fresh, literary-analytic perspective on the experience of anorexia and the burgeoning pro-anorexia cult.

*Wasted*, in particular, both illustrates and participates in the reading disorder phenomenon. In the next section I elaborate on the ways Hornbacher’s memoir offers a presentation of anorexia nervosa vulnerable to being read as positive and may, intentionally or not, influence certain readers to “try it” themselves. This will be followed by close textual analysis and exploration of the way Hornbacher’s own disordered reading suggests the reality of anorexic discourse or anorexia as a reading disorder. I conclude with a discussion of disordered reading attitudes in relation to the advent of the “pro-anorexia” cult.

## An Addiction to Starvation: Getting *Wasted*

When considering anorexia memoirs’ involvement in eating disorder acquisition, it becomes clear that not all anorexia life-writing is charged with the same “toxic” influence as *Wasted*. Prior eating disorder memoirs, particularly the earliest published texts, which appeared in the United States during the 1980s, do not seem to generate this type of criticism or attention at all. Other eating disorder memoirs such as Helena Wilkinson’s *Puppet on a String* or Joan Johnston’s *Feast of Famine*, and semi-autobiographical first-person works of fiction such as Deborah Hautzig’s *Second Star to the Right*, were not criticized (or praised) for being dangerous, triggering, or motivational.^27^ Indeed, *Second Star to the Right* advertises itself as a “moving, perceptive and gripping book . . . that all young people should read.”^28^ Thus it seems that not all acts of reading, or perhaps not all texts read, are as prone to the alternative interpretative processes involved in disordered reading. So what might explain this difference? *How* does disordered reading transform texts like *Wasted*, and what is it about those texts and not others (like *Feast of Famine*) that fit the disordered mode so well?[Other P-491]

One key difference between *Wasted* and its apparently harmless predecessors is that the majority of the earlier anorexia memoirs are heavily religious, and thus present the condition as a tragic affliction— that is, as utterly extrinsic to identity. Moreover, these texts portray recovery from anorexia as an often instantaneous product of faith or trust in God. In *Feast of Famine*, for instance, Johnston pinpoints her recovery quite literally to the minute: a religious epiphany reached while volunteering for a Catholic Apostolic charity.^29^ Hornbacher instead presents anorexia as an integral part of her identity and reveals her recovery, absent any *deus ex machina*, to be far less absolute or easily resolved—a precarious position, her world seemingly always in thrall to a persistent anorectic drive: “It’s never over. Not really. Not when you stay down there as long as I did, not when you’ve lived in the netherworld longer than you’ve lived in this material one, where things are very bright and large and make such strange noises. You never come back, not all the way” (285). Hornbacher’s position fundamentally transforms anorexic subjectivity; this establishment of anorexia as a *condition of being* marries anorexia nervosa to personality, seeing it less as an illness and more as an almost inevitable behavioral manifestation of global character attributes. In this way, whereas Johnston’s memoir depicts anorexia as a departure from virtue, an absence of faith, and as symptomatic of a profound lack of a higher power, purpose, or meaning, Hornbacher’s account of her journey to and tentative return from the anorexic “netherworld” reveals anorexia to provide meaning for enduring “anorexic” personality traits.

Indeed, not only does Hornbacher create room for understanding anorexia as an identity category, but also this category is subject to particularly persuasive rhetorical management. A large part of the criticism leveled at her purportedly cautionary tale of addiction concerns her consistent association of both the central behavior (barely eating) and the disorder as a whole (anorexia nervosa) with positive personality traits and beneficial social or emotional side effects, from ideas of purity and perfection to intelligence, social standing, and liberty, while also presenting the text as something “to keep people from going where I went.”^30^ Hornbacher’s perspective on anorexia is largely ambivalent and often reverential. She writes of her anorexia as pure, focused ambition (107); “success” (19); an addiction which vitalizes, making her “high as a kite, sleepless, full of a frenetic, unstable energy” (105): to Hornbacher, the anorectic is able to experience life as though standard human physiological limitations do not apply. She describes the “very, very intense” power of starvation (105). And while [Other P-492] the title, *Wasted*, immediately marries her depiction of eating disorders with the image of intoxication, and her further characterization of her experiences with anorexia and bulimia nervosa as addiction (on nine separate occasions) seems to point towards the harms of these endeavors, depicting her eating disorder as an addiction does not necessarily act as a warning. Rather, it downplays its seriousness, its life-threatening dangers. Hornbacher writes of her trial of and addiction to various narcotic substances in her early adolescence, including heroin, with a surprisingly low affect and blasé attitude (72); consequently, her subsequent addiction to starvation becomes similarly mundane. Certainly, Hornbacher equates the dangerousness of anorexia nervosa to the dangerousness of intravenous drug injection in the mid-1980s—but as she presents heroin use in a thirteen-year-old as mundane, so she leads the susceptible reader to understand anorexia as such.

Hornbacher also relates anorexia to intelligence and abnormally high scholastic achievement—“anorexia,” she writes, is a “more cerebral” form of eating disorder (202), and “people with eating disorders tend to be both competitive and intelligent. We are incredibly perfectionistic. We often excel in school, athletics, artistic pursuits” (136). Thus, anorexia is a more cerebral subset of an *already* perfectionistic, intelligent, incredible state of being: an elite, enviable endeavor. Hornbacher’s presentation of anorexia as a marker of distinction implies that, under competitive circumstances, the disorder is a way of setting oneself apart from the rest, as superlative—from “more” to *most*. Hornbacher’s writing of anorexia nervosa (literally) embodies the epitome of success. While for some readers the ability to identify her position here as non-normative will remain intact, Hornbacher’s favorable framing of her anorexia means that the possibility of remaining neutral and detached is a greater challenge, and assimilation to her values and identity the more likely consequence.

Whereas other writers have emphasized the involvement of religious belief in their recovery from anorexia, Hornbacher expresses concern about the tendency to surrender responsibility for illness and recovery to a higher power. Discussing the restrictive tenets of 12-step addiction programs, she writes: “There is a tendency to say: I have an addictive personality, I am terribly sensitive, I’m touched with fire, I have Scars. There is a self-perpetuating belief that one simply cannot help it, and this is very dangerous. It becomes an identity in and of itself. It becomes its own religion, and you wait for salvation, and you wait, and wait, and wait, and do not save yourself. If you saved yourself and did not wait for salvation, you’d be self-sufficient. [Other P-493] How dull” (131). Yet, though she disdains it, Hornbacher nevertheless participates in the processes she warns against. She applies a religiosity to her anorexic behavior, interpreting it in ecclesiastical terms and with a devotion commonly reserved for gods or saints. Hornbacher refers to her anorexia as a “crusade” on five separate occasions.^31^ Employing a religious vocabulary in this fashion highlights the author’s reverence for her eating disorder and suggests not only its intensity but also its merit: consistent devotion to one’s religion (or cult) and adherence to its doctrines of faith will be rewarded with eternal life. This transformation of addiction to devotion is a rhetorical device frequently employed by pro-eating disorder websites for this very reason. Hornbacher describes her desire for faith: “Had we a god, it might have been Dionysus. We, his followers, imagined ourselves maenads, half-believing in divine possession, half mocking it. . . . We wanted to be that genius, that idiot mad with the world of his mind. A thrum of self-destruction, and a joy all tangled up. . . . We were very hungry” (104). This passage reveals, in addition to the religious elements of her disorder, the equation of its obsessional, dysfunctional behaviors with productivity, a theme that reverberates throughout the memoir.

Hornbacher’s understanding of her achievements as the fruits of her deep entrenchment in her anorexia suggests that the disorder *per se*, along with her academic talents, is a success. Hornbacher here encourages her readers to covet her illness as well as envy her ability. The disordered reader feels that, unless she becomes as successfully anorexic, that is, *as sick as* Hornbacher—sleeps less, eats less, weighs less—and achieves more, she will fail. Hornbacher repeatedly underscores the hyperactive workaholic tendencies that accompany her anorexia. Relating the initial stages of the disorder, which occurred when she was aged fifteen and at boarding school, Hornbacher boasts, “I was running twenty-five miles a day on a knee that was beginning to split” (110). At seventeen: “I stopped sleeping. . . . I brought a stack of books and a notebook, packs of cigarettes, and sat coiled in a chair in the corner, rubbing my eyes, red in the almost tangible haze of smoke, swallowing coffee so thick it left sludge in the bottom of the cup. Reading Bertrand Russell and John Stuart Mill and Marx” (218). At nineteen she recalls sleeping less than three hours a night, “eating 320 calories a day” (247); “writing a weekly column” and “sidelining as a freelance research hack for a couple of papers” (248); “going to school full-time [and] pulling a 4.0 grade average” (248)—and weighing about 80 pounds.[Other P-494]

## *Wasted*’s Rhetorical Bent: The Art of Starvation

Hornbacher exploits an idiosyncratic writing style and her own disordered reading *of* her behavior to present anorexia nervosa as a positive condition. But my argument is not only that *Wasted* presents a non-normative reading of anorexia in which the condition is suffused with positive meaning and associations, but also that Hornbacher’s writing reveals her own disordered reading behavior. Exploring this phenomenon in closer detail provides a useful route to understanding anorexia as a reading disorder. I shall show how reading practices are involved in Hornbacher’s anorexia, and suggest that her expression and explanation of this (ideological as much as proximal) closeness between ways of eating and of reading *discloses* as well as participates in a peculiar and intimate relationship between anorexia and reading.

This section first addresses how Hornbacher’s distinctive writing style works to promote anorexia nervosa in the disordered reader; it then turns to Horbacher’s testimony of her own disordered reading to discuss the way she ties together reading and anorexia. Finally, I explore the implications of my method for understanding reading disorders and anorexic discourse. Here I must state explicitly that I do not believe *Wasted* was written as an intentionally pro-anorexic text. It is not the aim of this paper to apportion blame to Hornbacher (or her readers) for their involvement in these rhetorical processes; the hope is to shed some light on this hitherto little-explored relationship between reading behaviors, narrative engagement and anorexia nervosa. *Wasted* and Hornbacher have a central role in this epiphenomenon but it is unlikely to have been a deliberate or welcome one. Indeed, responding in an interview to a question about the intended audience for the book (a question which also suggested the memoir was harmful), Hornbacher said, “I will honestly say I did not write this for people with eating disorders. . . . Did I write it to help others? Yes. I wrote it so their mothers, husbands would put them in the hospital.”^32^ She states in the introduction to *Wasted* that she wrote it “to keep people from going where I went” (7), yet her prose seems at odds with these apparent intentions: she consistently addresses the reader as a confidante, a fellow anorectic—beckoning to readers with her extensive use of the second person (“you have been removed from the world” [145]; “you can subsist a long time, eating just a little” [268]) and confirming a shared identity by using “we” addresses throughout (“we have to choose sides” [154]). I believe the necessarily introspective direction of memoir conspired with what I shall show is her own disordered [Other P-495] reading attitude to render her naïve to its potential to harm readers. Since Hornbacher’s writing—in style as much as in substance—reveals her to be a disordered *reader*, the potential interpretation of her words as promotional may not have seemed possible or plausible, or have occurred at all to her during the writing process.

This apparent gulf between the expression and reception of memoir information emerges in the myriad foibles in Hornbacher’s writing. What may be self-conscious attempts to locate herself at some remove from her more graphic accounts of illness emerge as mechanisms inviting readers to take on an anorexic subject position. One such example (a surprising feature of a memoir) is Hornbacher’s prolific use of the second-person rather than first-person narrative. Her first use of “you” occurs just thirteen pages into her first chapter, and increases in frequency and duration as Marya’s eating disorder progresses.^33^ Some of these passages work to induce a “disordered” identity in the reader. For example: “The problem in your life is your body. It is defined and has a beginning and an end. The problem will be solved by shrinking the body. Contain yourself” (42), and “You will lift the toilet seat, carefully slide your fingers inside your mouth and down your throat, and puke until you see orange” (61). Ordinarily, being forced to assume such an identity is alienating, foreign, uncanny; it coaxes from readers a dysphoria between mind and body, a corporeal sense of wrongness. A reader made to throw up vicariously through a text will be, like Marya’s (non-disordered) friend who tried it, “gripped . . . by a sudden sense that what [they are] doing [is] *wrong*. Not wrong in the sense of sinful, but wrong in a human sense—a crime against nature, the body, the soul, the self” (123). Yet since Hornbacher consistently, almost conspiratorially, invites her readers to identify with her, passages such as these have no room for alienation. Hornbacher’s prose thus skillfully guides the reader towards painlessly, perhaps even imperceptibly, synthesising her own thought patterns, beliefs, and perhaps even behaviors.

Throughout the memoir, Hornbacher takes pains to characterize herself as a “better” anorectic than anybody else in a combination of ways: the duration of her illness; her apparently superhuman abilities to subsist—or supersist—higher and better than mere mortals—without food or sleep; her alarmingly low weight (fifty-two pounds) and, perhaps most pertinently, her assumption of an authoritative writing style. Hornbacher is a self-made expert on eating disorders. Her academic mode of writing is unusual for a layperson, but especially so in a memoir, where footnotes, citations, and bibliographies are practically [Other P-496] unheard of. Her application of these markers of authority reinforce the impression that she really is the best at what she does—starving. Or, starving and remaining able to “[tap] out a virulent argument against Kierkegaard” in an evening and on an empty stomach (256). Hornbacher’s academic approach to writing her memoir reframes her own authorial role as educating more than confessing and reclassifies *Wasted* as textbook more than autobiography. Moreover, her upending of conventions of memoir constitutes a non-normative and disruptive approach to writing: as much as Hornbacher’s memoir lends itself to, invites, and participates in disordered reading, it is also itself an example of disordered writing. By this phrase I do not aim to haphazardly diagnose, much less disparage, literary or stylistic innovation per se. I invoke “disordered” in Eve Sedgwick’s sense of disarticulating or disrupting—as an invitation to see Hornbacher’s style as a strategic misalignment of expectations and conventions simultaneously illustrative of and participatory in the complex narrative performance of her anorexia.^34^

Hornbacher’s writing suggests that, if she is not deliberately styling the text as a how-to guide for eating disorders, she nonetheless has some consciousness of her role as teacher: the employment of “you” in most of her depictions of anorexic episodes does more than instruct the disordered reader to identify with her at her most ill moments; it instructs the reader to imitate them. Marya’s stay at Methodist is written with six pages of uninterrupted second-person narrative; her relapse in California covers five pages.^35^ She directs the reader, in unfaltering imperatives, to “step onto the scale each day, ten times a day. . . . When you wake up, when you get home from school, after you binge, after you purge, when you eat dinner, after you throw up dinner . . . before you gulp handful of laxatives, after they take their hideous effect” (162). Hornbacher’s instructions become more and more explicit as her memoir and disorder progress. By the time she reaches her lowest weight of fifty-two pounds, her writing moves away from implied directions to overt instructions. To the non-disordered reader, a passage of this nature would induce shock and perhaps disgust; for those whose reading is disordered, however, Hornbacher’s words will be taken at face value:

I’d read the same sentence over and over, to prove that I could sit in front of food without snarfing it up, to prove it was no big deal. . . . Try this at home, kids, it’s great fun. You take the edge of your spoon and run it over the top of the yogurt, being careful [Other P-497] to get only the melted part. Then let the yogurt drip off until there’s only a sheen of it on the spoon. Lick it—wait, be careful, you have to only lick a teeny bit at a time, the sheen should last at least four or five licks, and you have to lick the back of the spoon first, then turn the spoon over and lick the front, with the tip of your tongue. Then set the yogurt aside again. Read a full page. . . . Repeat. Repeat. Repeat.

(255)

That is, the disordered reader really will (at least want to) “try this at home.” What this passage also illuminates, in addition to the author’s increasingly disordered mode of writing, is Marya’s own disordered mode of reading—reading food, reading eating behavior, (not) reading consequences.

Hornbacher’s testimony reinforces the notion of “reading disorders” as a precursor to eating disorders and complicit in the establishment and perpetuation of anorexic discourse. Hornbacher’s presentation of her disordered eating behaviors in lengthy, excruciating (or, depending on the reader, exhilarating) detail, with little mention and less emphasis on the negative physical or psychological effects thereof, is an exhibition of her own disordered reading of the world and herself. Moreover, her understanding of anorexia as superior to bulimia, as a choice she made—after reading a book she felt encouraged her—and her general approach to reading and textual consumption from childhood on arrestingly illustrate the idea of a genuine and meaningful relationship between dysfunctional reading and dysfunctional eating.

Hornbacher was bulimic for six years before developing anorexia, and alternated between the two for some years. She presents the disorders as startlingly different: to Hornbacher, anorexia and bulimia exist, it appears, in different people. “Bulimia acknowledges the body explicitly, violently. It attacks the body, but it does not *deny*. It is an act of disgust and of need,” Hornbacher explains, depicting it as a gluttonous, excessive condition (93). Instead of a bulimic, she writes, “I wanted to be an anoretic. I was on a mission to be another sort of person, a person whose passions were ascetic rather than hedonistic, who would Make It, whose drive and ambition were focused and pure, whose body came second” (107).^36^ Her interpretation of her eating disorders thus reinforces the overall presentation of anorexia as elite. Crucially, alongside this, she suggests that her anorexia was something she “chose” after reading psychologist Steven Levenkron’s novel *The Best Little Girl in the World*, whose anorexic protagonist is, to Marya, “perfectly pure” (6, 43). She claims that she “decided that [Other P-498] . . . [she] would be an anoretic when [she] grew up” (43). Hornbacher’s rigid understanding of anorexia as superior to bulimia, and her belief that she developed anorexia—cultivated it, even—in response to reading about it, strengthens the hypothesis of a “reading disorder” that characterizes a predisposition in some people to eating disorders.

Girding this idea further is the prominence that books and reading have in Hornbacher’s memoir. These function as an obsession and emotional crutch just as much as her eating disorder does, a fact which betrays her reading *as* a disorder. She describes herself as being “perpetually grief-stricken” when, as a child, she finished books, which she read compulsively: “the book was dead. . . . What was the use? Why bother dragging the weight of my small body down to dinner? Why move? Why breathe? The book had left me, and there was no reason to go on” (28). At Interlochen, Marya accompanies her obsessive twenty-five-miles-a-day exercise habit with a book in hand, as though “reading” and “disorder” are inextricable entities, no longer discrete categories—each obsession complicit in the other (110). Indeed, as she attends college locally at the age of seventeen, Marya fuels her body and her disorder with books and seemingly little else. Recalling the “stack of books” feeding her in her nocturnal starvation behavior, her reading “Bertrand Russell and John Stuart Mill and Marx” to occupy herself in place of eating, Hornbacher’s attitude here and elsewhere suggests a cyclical relationship between her (disordered) reading habits and disordered eating habits: one makes the other worse and so on, ad nauseam (218).^37^

Whilst an inpatient at Lowe House, Hornbacher recalls being “fed” with books as well as food, using reading—which she does “voraciously”—to alleviate her anxieties (196). Indeed, no longer able to indulge her obsessional starvation, Hornbacher describes her disorder in relation to books in place of food: “We talked about books. They brought me books. I sat at the table, behind my battalion of books, peering over the top, half-reading, half-talking to them, telling them about my books. Then Staff took away my books” (198). These actions recall the once-popular and later widely maligned “rest cure” pioneered by late nineteenth-century American physician Silas Weir Mitchell. This treatment, aimed primarily at women, not only required total bed rest and the imposition of a rich, heavy, and large diet (indeed reminiscent of many anorexia refeeding programs), but also prohibited forms of mental exertion like reading and writing.^38^ Many prominent intellectual women were subjected to this treatment, including Virginia Woolf in England and Charlotte Perkins Gilman in the United States.^39^ Gilman, [Other P-499] who like Hornbacher describes having read “eagerly” and “greedily” as a child, recounts in her 1935 autobiography the prescription given to her by Mitchell to “have but two hours’ intellectual life a day. And never touch a pen, brush or pencil as long as you live.”^40^ This prohibition, she explains, is singularly painful to her: “to lose books out of one’s life, certainly more than ninety per cent of one’s normal reading capacity, is no light misfortune.”^41^ Indeed, for Hornbacher, too, it is at just this point—bereft of her ability to starve and her ability to read—that she states she “lost it altogether” (198). It is of interest to note that Hornbacher’s disorder is very much connected to the *act* of reading, as opposed to the content per se, or the physical object. Reading is disordered; being read to is not: she writes of being read to at Lowe House in wholly therapeutic terms; how, despite her resistance, “the reading set off tiny explosions of longing in [her] chest” (201).

## Anorexic Discourse: Pro-Anorexia and the Cycle of Disordered Reading

This final section seeks to examine how disordered reading and writing practices relate to the advent and growth of pro-anorexia culture and to illuminate the processes of anorexic discourse through looking at some of the rhetoric of pro-eating disorder websites.

Here I return, momentarily, to the beginning of Hornbacher’s illness and her decision to become anorexic after reading *The Best Little Girl in the World*. This event not only suggests that because she chose anorexia after reading the story of a “perfectly pure” anorectic (43), so too can (and should) the reader choose to be anorexic after reading Hornbacher’s story, it also offers the most compelling evidence for the notion of disordered reading and its role in anorexic discourse. Marya, as a disordered reader, is triggered to develop anorexia after reading an account of it she interprets as attractive; other (disordered) readers have read Hornbacher’s book and authored websites that encourage others (deliberately or not) to develop eating disorders. That is to say, not only are there disordered readers, those readers may also become disordered writers—like Hornbacher, and like the authors of pro-anorexia websites. It is my hypothesis that these websites are, in general, authored by those who read maladaptively in the fashion described above and who, consequently, showcase their personally disordered interpretations of text—from *Wasted* to images of the Holocaust—and, in so doing reinforce the validity of reading in this way.^42^[Other P-500]

As touched on previously, pro-anorexia pages tend to reconsider the disorder as a lifestyle, usually a pious, ascetic way of life, consumed by devotion to their goddess, Ana.^43^ One prominent tenet of the “faith” is the “Thin Commandments,” a list of doctrines that state overtly the claims Hornbacher only implies. For instance, “3. You must buy clothes, cut your hair, take laxatives, starve yourself, do anything to make yourself look thinner”; “10. Being thin and not eating are signs of true will power and success.”^44^ The near universal presence of this list across pro-ana websites is of particular interest when considering pro-anorexia as a symptom of disordered reading. The Thin Commandments were not written as an instructive basis for the pro-ana cult. Rather, they were conceived of by eating disorder therapist Carolyn Costin as a tool to use in the treatment of anorexia.^45^ The repurposing of this initially therapeutic text into an instructive model for inspiring anorexia that has become cornerstone of the pro-anorexia movement demonstrates both the disordered reading and the discursive process it encourages. The act of appropriating and reproducing texts across sites perpetuates an anorexic discourse by effacing the therapeutic intent and cementing the disordered reading as the dominant narrative.

These websites also commonly feature themes of rebirth, transformation or resurrection, adopting images like dragonflies or butterflies (*Blue Dragonfly*; *Cerulean Butterfly*) as emblematic of the anorexic endeavor—to transcend. On their totem, Blue Dragonfly offers the statement that “[d]ragonflies are above ordinary, amazingly skilled at what they do. Likewise, it takes much skill and determination to starve yourself and exercise all the time. Dragonflies to me are ‘perfection,’ the way they change and improve, as we strive to perfect ourselves.”^46^ In a similar vein, other sites offer detailed, aspirational descriptions of the goddess figure, Ana, depicting her as a small, fairy-like creature with blue eyes and blond hair. To Ana devotees, she is a reminder of their perceived shortcomings: “She’s someone who’s perfect. It’s different for everyone—but for me, she’s someone who looks totally opposite to the way I do.”^47^ Ana, this superlative idol, thus emerges as an ideological doppelgänger of Hornbacher, who exists as a god in her memoir, simultaneous author and lead character of the anorexia bible.

The multimedia aspect of the Internet redoubles these websites’ influential potential. Whereas a simple text can only go so far, websites can couple text and image, and use video, even music, to promote anorexia to readers, sometimes outside of the bounds of the community: Video-sharing website YouTube currently hosts over 37,000 “thinspiration” videos. “Thinspiration” is usually found in images of [Other P-501] extremely thin women, often celebrities and supermodels although with an increasing focus on “real girls,” frequently digitally altered to appear slimmer.^48^ These pictures depict severely emaciated women and men. Many are featured in memoriam, showing death dates and/or death weights.^49^ These images do what Hornbacher’s memoir cannot: they articulate the apogee of “successful” anorexia nervosa—they literally show *the picture of death*. One such image shows the corpse of a woman slumped over a toilet with the caption “She was 19 and had anorexia and bulimia for 5 years. Died at 5’ 1” (155cm) and 94 lbs (43kg) after her stomach ripped after eating 5.6 liters . . . of food.”^50^ Although this caption purports to be a warning against the dangers of anorexia, it actually serves the disordered reader as a warning against the dangers of eating. It was not, after all, starvation that killed the woman but, rather, consumption: her perceived gluttony. The written message overwrites the visual, subverting the stated intention and proffering to the reader an alternative narrative more concordant with a pro-anorexia stance. Moreover, positing photographs such as these as apparent warnings against eating disordered behavior is entirely duplicitous—not only is the impact of the photo very different from its alleged intentions, hence its application as a warning spurious, it is also an entirely impotent tool in the treatment of eating disorders, and the astute reader may recognize this tacit assumption as the product of a culture persistently beholden to the myth of anorexia as having a fundamental visual basis. Again, visual culture is given too much credit: no photo can cure anorexia—not even, in perhaps the most shocking use of images on pro-eating disorder websites, where Holocaust imagery is juxtaposed with photographs of anorexia sufferers.^51^

These websites promote eating disorders, intentionally or not, solely on account of the authors’ maladaptive interpretation of image and text: disordered representation and reading. The publishing of images of corpses; the memorials; the equation of anorexia and the Holocaust; “religious” doctrine—all betray the authors’ own disordered approach to reading and *their* transformation of texts into promotional materials. The websites, consequently, saturated with maladaptively interpreted documents, serve to endorse reading disorders, as Hornbacher’s text also does, thereby perpetuating an anorexic discourse and the exploitation of previously value-neutral texts as encouragement.[Other P-502]

Noting the current fashionable status of anorexia nervosa and the advent and proliferation of websites promoting the anorexic lifestyle, this essay has sought to elucidate the phenomenon. The presence, prominence, and popularity of this emergent discourse, which frustrates a variety of dominant medical, psychiatric and cultural narratives, is greater than ever. Further, a growing body of research orbits around the concern that anorexia nervosa might be transmissible through secretive online networks—and highly contagious.^52^ Offering a literary-analytic perspective on the idea of anorexia as communicable through texts, I have presented a possible underlying mechanism for the phenomenon. Proponents of “Ana” prescribe, among other things, reading “triggering” material, the ultimate of which is widely considered Marya Hornbacher’s memoir *Wasted*, which has been accused of glamorizing the illness and encouraging readers to “try it.” Coupled with knowledge of research that shows starvation in healthy men induces eating-disordered behavior, and that such behavior often focuses on obsessive acts of reading, this paper outlines a hypothesis that states a relationship between disordered modes of reading and disordered eating attitudes and behaviors.^53^ Exploring the rhetoric Hornbacher exploits in her memoir illuminates how those predisposed to anorexia might interpret the text. These readers, for whom anorexia memoirs and other eating disorder literature hold special meaning, consume texts in unexpected ways—one example being those passages that, though they would alienate and appall the majority of readers, act as instructions for those who have non-normative reading attitudes—and regurgitate these interpretations across networks of similar readers online. Close reading of pro-anorexia web content allows me to speculate about how disordered reading works on the other “end” of text—not only as an illustration of individual reading and eating pathology, but as a mode of reception. Excavating Hornbacher’s text clarifies some of the specific ways in which *Wasted* may encourage disordered eating; further, episodes in her memoir emphasize her own fixation with analysis in general and book-reading in particular. Those textual moments suggest that she herself reads in this distinctive disordered way and that authors of pro-anorexia websites also have these disordered reading attitudes, suggesting, further, that the maladaptive analytical process of a reading disorder necessarily involves the proliferation of such processes—forming an overarching rhetoric of disease; a pernicious cycle in which self-destruction is, accidentally-on-purpose, encouraged: an anorexic discourse.[Other P-503]

This brief analysis of narrative and anorexia has revealed that reading, writing, consuming, constructing, or deconstructing texts each holds a significant role in anorexia nervosa: there is an intriguing interplay between these ways of engaging with narrative and the instigation, development, maintenance, and transmission of disordered eating behavior. Further investigation ought to be initiated to better understand how anorexia nervosa is narratively constituted, transmitted, shaped, and performed and how narrative engagement and anorexia interact. The close reading of life-writing in relation to anorexia acquisition and the illness experience would begin to address questions such as: What makes anorexia so narratively compelling? What can be learnt about treating and preventing anorexia from how people who become anorexic retrace and explain that becoming? What might reading across anorexia memoirs reveal about anorexia as a “reading disorder”? Are there ways of reading and approaching texts indicated in these narratives that are indicative of future illness and illness trajectories? And how, for example, do shifts and differences in anorexia illness experience within and between narratives, especially life-writing, provide a means to understanding anorexia treatment and recovery?

## NOTES

1 Brumberg, *Fasting Girls*, 12.

2 Showalter, *Female Malady*, 293. Emphasis added.

3 Reaves, n.p.

4 See “Thin Commandments”; “Ana’s Law”; “Letter to Ana.”

5 Johns, 10.

6 See “Proceed with Caution”; Swerdel, “Where to Begin”; Johnson, “‘Wasted’ Is Not Constructive.”

7 Hornbacher, 43.

8 Ibid., 7.

9 De Quincey, 1, 63; Hayter, 105; Berridge and Edwards, 53.

10 See Ellmann, 79; Horn, 133. See Kennaway, “Two Kinds.”

11 See “Mirror Mirror” and “Looking Glass.”

12 See Crisafulli et al., 338.[Other P-504]

13 See Fleming and Szmukler; Brotman, Stern, and Herzog; Crisafulli et al., 333.

14 See Roehrig and Maclean.

15 See Crisp, 148.

16 See Brandenburg and Andersen.

17 See Becker et al.; Cachelin et al.; Gordon, Perez, and Joiner.

18 See Treasure and Russell.

19 See Gremillion, 10.

20 Other men consumed up to forty packets of chewing gum per day (until, consequently, access was restricted); others stole food, or took it from garbage bins, to binge on, only to feel intensely guilty afterwards. One participant noted in retrospect that “eating became a ritual . . . some people diluted their food with water to make it seem like more. Others would put each little bite and hold it in their mouth a long time to savor it” (quoted in Kalm and Sembla, 1349). See Ball.

21 See Harper, Sperry, and Thompson.

22 See Bardone-Cone and Cass, 537.

23 See Thomas et al.

24 Ibid., 422–23.

25 Ibid, 423.

26 See Kalm and Sembla.

27 See Wilkinson, *Puppet on a String*; Johnston, *Feast of Famine;* and Hautzig, *Second Star to the Right*. Hautzig’s book is actually closer to a mock-fictional first-person work of confession, though it is billed as fiction. First published in 1981, the 1998 United States reprint of *Second Star* featured a new afterword by the author detailing—graphically—the horrors of her own long struggle with anorexia nervosa (which, like Hornbacher, she describes as an addiction). See Hautzig, “Second Star to the Right: Afterword.” Triggers have received increased media attention of late; here the idea of “triggering” relates to the idea that particular sensitive content (for example, in the case of anorexia, detailed descriptions of behaviors, low weights achieved, or calories [not] consumed) may affect vulnerable readers (including recovering or recovered anorexia patients) in a way that is psychologically detrimental.

28 Hautzig, inside cover.

29 See Johnston, 210.

30 Hornbacher, 7. Subsequent page references in body of text.

31 See Hornbacher, 48, 64, 109, 110, 134.

32 See Fallon; Hornbacher.

33 I use the first name Marya here and throughout to make a distinction between Hornbacher as author and (Marya) Hornbacher as written as a “character” within the memoir.

34 See Sedgwick.

35 See 145–50; 160–64.

36 Hornbacher uses the noun “anoretic” over the more standard “anorectic.”

37 Hornbacher makes an implicit value judgement when she deems some texts “anorexia” books (versus texts that are gluttonous and indulgent—those she considers “bulimia” books). Hornbacher describes her childhood reading in a similarly obsessive way, but while she is bulimic her textual consumption is far less dry, less academic and much more an emotional crutch. Other writers have taken a similar position on the *types* of reading that relate to eating disorders. For example, Sheila MacLeod’s 1981 memoir *The Art of Starvation* describes the crucial difference between “junk books” (which she explicitly compares to “junk food”) and the sorts of books and reading behaviors that she recognizes as making her “predisposed to become anorexic” (MacLeod, 42).

38 See Sicherman.

39 See Poirier.[Other P-505]

40 Gilman, 99; 96.

41 Ibid., 100.

42 Many pro-eating disorder web communities do not state explicitly their purpose (out of fear of being shut down) and will, frequently, state that they are *not* promoting anorexia even when everything on the site suggests otherwise. Consequently, this section will examine a selection of pro-eating disorder web pages irrespective of any disclaimers that may deny it: such denials are no deterrent, just as Hornbacher’s contradictory claims about her intentions for *Wasted* “to keep people from going where I went” are not (7).

43 Owing to the controversial content of these websites, they are prone to being shut down or moving web hosts. So that the resources quoted remain accessible, I have quoted stable URLs from the Web Archive throughout.

44 “Thin Commandments,” *Ana Handbook*.

45 Costin, 13–14.

46 See “What BlueDragonfly Means.”

47 See “Pro-anorexia Movement.”

48 For example, “Real Thinspo!”

49 For example, “Jeremy Gillizter.”

50 See “Bulimia Kills: Warning.”

51 See “Starvation.”

52 See Lavis; Maloney; Starcevic and Aboujaoude.

53 See Kalm and Sembla; Ball.
